# Photonic crystal biosensor featuring an eye-shaped cavity for precise identification of cancerous cells

**DOI:** 10.1038/s41598-025-07938-y

**Published:** 2025-07-04

**Authors:** Salma Rizk, Seham Abd-Elsamee, El Said A. Marzouk, Nihal F. F. Areed

**Affiliations:** 1 Department of Electronics and Communication Engineering, Faculty of Engineering, Mansoura University, Mansoura, 35516 Egypt; 2 Communications Department, Delta Higher Institute for Engineering and Technology, Mansoura, 35516 Egypt

**Keywords:** Photonic crystal (PhC), Biosensor, Refractive index (RI), Cancer detection, Sensitivity, Q-factor, Cancer, Optics and photonics

## Abstract

This article presents a highly sensitive and thermally stable photonic crystal (PhC) biosensor designed for accurate cancer-cell detection. The proposed sensor features a square-lattice of silicon rods (radius 0.1 µm) with a photonic bandgap spanning 1.2–2.1 µm. It includes two line-defect waveguides for input and output, and a uniquely engineered Eye-shaped cavity that holds the analyte as embedded rods. These rods are strategically arranged along the Eye-shaped boundary and the central area resembling an iris, facilitating accurate detection through resonance wavelength shifts triggered by changes in the refractive index. The biosensor demonstrates excellent transmission efficiency (69.7%–99.9%), high sensitivity (236–243 nm/RIU), and a strong quality factor (15,764–87,070), ensuring sharp and clearly defined resonance peaks. A key advantage of the design is its linear response to refractive index variations, which enhances detection accuracy and supports reliable real-time biosensing. Moreover, the sensor maintains stable performance across a wide temperature range (25 °C to 75 °C) and exhibits robust tolerance to fabrication variations. These features validate the biosensor’s precision for biomedical diagnostics.

## Introduction

Cardiovascular diseases, cancers, diabetes, and respiratory diseases account for more than 60% of all deaths worldwide.^1^. Cancer is an illness characterized by abnormal and uncontrolled cell growth. Common types include cervical cancer (HeLa), skin cancer (Basal), two breast cancers (MDA-MB-231 and MCF-7), blood cancer (Jurkat), and adrenal gland cancer (PC12)^2–5^. A well-known traditional cancer analysis requires considerable time, money, and large-volume blood samples for detection.^6^. New biomedical research focuses on innovating Lab-On-A-Chip biosensors for quick and precise outcomes^7^.

Recent advances in optics and photonics demonstrate the rapid evolution of the field and its potential to open the door to new sensing technologies. One of the most promising areas is photonic crystal-based devices, which can control light with great precision and have shown strong potential in sensing applications. Refractive index (RI)-based sensors are valued for their high sensitivity, real-time detection, and versatility. They have been used to detect organic compounds, hazardous chemicals.^8^. biomolecules, pathogens^9^, pressure ^10^,, temperature ^11^, glucose concentration^12^ and flow in microfluidic systems. ^10^ This broad applicability highlights the importance of customizing RI sensors for specific needs, supporting the development of the photonic crystal-based biosensor introduced in this work. Biosensors are very sensitive and can detect small amounts quickly. They have a compact design, are measured in microns and nanometres, and can be integrated with optical circuits. (OICs) ^13^. One of these biosensors is PhC biosensors. PhCs are made up of regular patterns of materials that either contain electric charges (dielectric) or metals (Metallo-dielectric) with varying dielectric constant materials in different values. ^14^. These biosensors are available in different forms, including one-dimensional (1D), two-dimensional (2D), and fibre-based configurations. They are widely utilized in resonators. ^15^, waveguides ^16^, fibres ^17^, switches ^18^ and biosensors ^19^. These structures are designed to influence the electromagnetic wave propagation within them. Due to this regular pattern, light transmission is obstructed in specific ranges of frequencies, creating a Photonic Band Gap (PBG) ^20^. Defects occur in these repeated structures, the periodicity is entirely disrupted, allowing for control and manipulation of light. ^21^ . 1D PhCs consist of regularly spaced layers with varying refractive indices, creating a bandgap that allows specific wavelengths of light to pass through ^22^. In biosensing, introducing a biological substance alters the optical properties, causing shifts in the bandgap or resonant wavelength, which can indicate the presence or concentration of specific analytes ^23^. These biosensors enable label-free detection of biomolecules like DNA and proteins, offering a simple design that suits various sensing applications ^24^. 2D PhCs are being widely studied for designing optical devices due to their ability to confine light better, calculate PBG efficiently, control spontaneous emission effectively, and integrate with other devices more easily than 3D PhCs ^25^. PhC biosensors quickly and effectively identify proteins ^26^, DNA ^27^, Diabetes ^28^, or alternative biological substances for disease detection. Cancer cells demonstrate a greater refractive index compared to normal cells because of their elevated protein levels in the cytoplasm. Therefore, detecting the difference between healthy and cancerous cells is important to create an effective diagnostic sensor ^29^. The difference in RI between healthy and abnormal tissues shifts the resonance frequency in the biosensor’s optical transmission spectrum ^30^. A high-quality factor (Q) significantly changes the transmission spectrum due to a minor alteration in the refractive index (RI). In designing and evaluating biosensors, there is a trade-off between key performance metrics such as sensitivity, quality factor (Q-factor), detection limit (DL), figure of merit (FoM), and power efficiency. Achieving optimal values for one metric can sometimes lead to compromises in others, as in ^31^. The reported design in ^31^ is based on a refractive index sensor designed with a GaAs rod structure that demonstrated promising results for malaria detection. The results show that the biosensor has a high sensitivity of 798.143 nm/RIU, a high Q-factor of 9881.926, and a high FoM of 4496.079 RIU-1. However, the sensor showed limitations in terms of DL and power efficiency. Additionally, the design in ^32^ is based on a Silicon-On-Insulator (SOI) platform designed to detect cancer cells. It is focused on achieving high sensitivity to differentiate between similar analytes effectively. Yet, they may exhibit reduced DL and Q-factor. Other engineered configurations ^33–35^ utilize a circular resonator nanoring, emphasizing the enhancement of the Q-factor instead of sensitivity to generate clear resonance peaks. Furthermore, a higher Q-factor minimizes signal noise, resulting in more dependable and stable measurements over time. In ^6^, Researchers developed a biosensor to identify various blood components. The design in ^36^ is based on a hexagonally structured PhC biosensor to detect various types of cancer cells. This structure exhibits a remarkable quality factor, acceptable sensitivity, a higher figure of merit, and a positive shift in resonant wavelength with values of 1741, 72.28 (nm/RIU), 70.74 (RIU^−1^), and 1.04, respectively. The researchers in ^37^ designed a 2D microcavity ring with a waveguide structure to sense the existence of malignant cells in the analyte. They achieved the highest sensitivity with a value of 995 nm/RIU and a low Q-factor with a value of 70. While these studies demonstrate significant advancements in PhC biosensor design, they primarily focus on circular ^31^, hexagonal ^36^, or microcavity-based resonators ^37^, with limited exploration of defect-based structures, particularly elliptical defects. Elliptical-shaped defects ^32,35,38^ offer distinct advantages in manipulating the photonic bandgap, enhancing light confinement, and improving sensor performance regarding Q-factor and sensitivity. Additionally, the placement of input and output ports plays a crucial role in optimizing coupling efficiency ^32–34^. Incorporating the elliptical defect shape and strategic port positioning, this design principle played a crucial role in shaping our biosensor configuration.

In the paper presented, a two-dimensional PhC biosensor featuring an Eye-shaped defect has been developed to distinguish between various types of cancer cells, including blood cancer (Jurkat), skin cancer (Basal), cervical cancer (HeLa), two forms of breast cancer (MCF-7 and MDA-MB-231), and adrenal gland cancer (PC12). The refractive indices differ according to the type of tissue, which is an important factor in optical biosensing applications. These cancerous and model cells exhibit refractive indices ranging from 1.36 to 1.401, making them ideal candidates for PhC, designed to identify cancer cells by taking advantage of the optical property differences between normal and malignant cells^39^. Accurately measuring these indices allows for highly sensitive, label-free cancer detection, which is essential for early diagnosis and monitoring of treatment. The performance indicators (Q-factor, Sensitivity, FoM, and power efficiency) are calculated from the transmission spectra. The results show a higher Q-factor and FoM with acceptable sensitive PhC biosensor values.

## Proposed structure and operation

**Fig.1:**
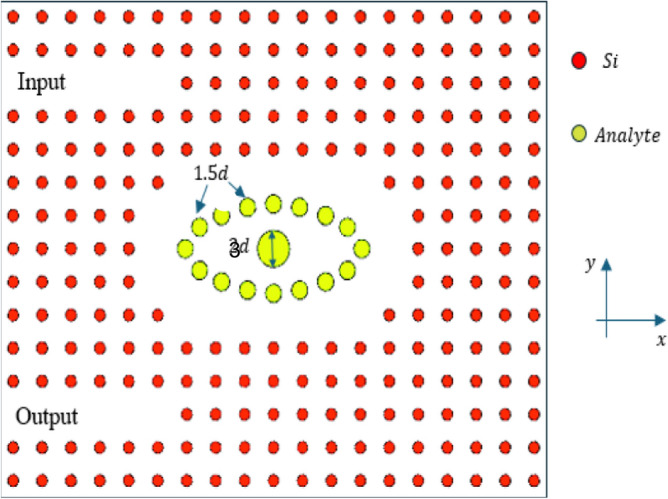
Schematic diagram for the proposed biosensor.

The Eye-shaped cavity-based 2D PhC biosensor is illustrated in Fig. 1. The configuration features a rectangular arrangement (21 × 17) of circular silicon rods (n_Si_ = 3.46) set within an air medium, with a lattice constant of Λ = 540 nm. Each rod measures a diameter, *d,* of 200 nm. The optimized geometric parameters for the suggested 2D PhC biosensor are detailed in Table 1, based on the analysis presented in the results section. As shown in Fig. 1, the design features two linear defects functioning as waveguides: one for sending the optical signal and the other for its reception. Furthermore, an Eye-shaped cavity is incorporated, designed to hold the analyte that needs to be detected, enabling accurate sensing through changes in its refractive index from 1.36 to 1.401.

**Table 1:** Geometric parameters of the optimized biosensor.

Geometrical Parameters	Dimension (µm)
Length of PhC platform	11
Width of PhC platform	8.85
Lattice constant, $\Lambda$	0.540
Si-rod diameter,$d$	0.2
Eye-shape-defect diameter	$1.5d$
Iris rod diameter	$3d$

A two-dimensional (2D) Finite Element Method (FEM) was employed to simulate and optimize the proposed design through calculating the transmission spectra. The resulting spectra are subsequently analysed to determine the following metrics: resonant wavelength, Q-factor, S, FoM, and power efficiency (η). These metrics can be defined as follows:

Q-factor is a unitless quantity and measures how selective the sensor is. A sharper, resonant peak is better for improving the quality factor.^40^. Q-factor can be calculated using Eq. (1):

1 $${\text{Q}} = \frac{{\lambda_{0} }}{{\Delta \lambda {\text{FWHM}}}}$$

where λ_0_ is the resonant wavelength and Δλ_FWHM_ is the total width at half-maximum for the primary transmission spectrum.

Sensitivity (S) refers to the proportion of the change in the resonant wavelength resulting from variations in the refractive index.^40^. It can be calculated from Eq. (2).

2 $${\text{S}} = \frac{{{\Delta }\lambda }}{{{\Delta n}}}$$

where Δλ is the change in the resonant wavelength and Δn is the variation in the refractive index.

The figure of Merit (FoM) helps in the determination of the smallest change detected in optical biosensors.^40^ And can be calculated using Eq. (3).

3 $${\text{FoM}} = \frac{{\text{S}}}{{{\Delta }\lambda {\text{FWHM}}}}$$

where *S* is the sensitivity and Δλ_FWHM_ is the total width at half-maximum for the primary transmission spectrum.

Power Efficiency (η) is a measure of how much energy is being used effectively to do work, compared to the amount of energy that is being wasted^40^ And can be calculated using Eq. (4).

4 $$\upeta =\frac{{\text{p}}_{\text{out}}}{{\text{p}}_{\text{in}}}\times 100$$

where p_in_ and p_out_ are the input signal power and the output signal power.

## Results and performance analysis

**Fig. 2:**
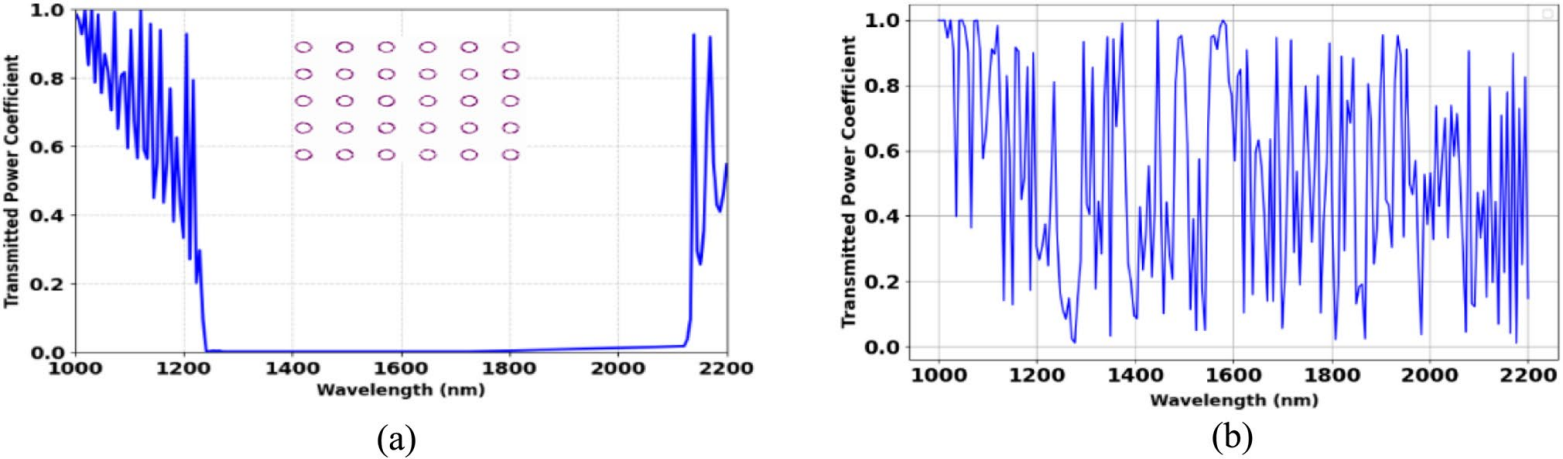
(**a**) Transmission spectrum of the considered photonic crystal platform without defects for TE Mode, (**b**) Transmission spectrum of the considered photonic crystal platform without defects for TM Mode.

The results section starts with the evaluation of the transmission spectra for the defect-free PhC platform, which facilitates the estimation of the photonic bandgap. Next, a parametric investigation is performed to identify the optimal structural dimensions that yield the highest sensitivity and Q-factor. The outcomes of the biosensor designed with these optimal dimensions are then presented and compared with existing literature to emphasize its importance. Initially, the photonic bandgap (PBG) of the utilized PhC platform is determined, which serves as a crucial step in the design of the suggested biosensor. The 2D PhC structure is composed of a 21 × 17 array of silicon rods and organized in a square lattice. The transmission spectra for the TE and TM modes exhibit distinct bandgap characteristics, as illustrated in Figs. 2(a), (b), respectively. The TE mode demonstrates a well-defined photonic bandgap within the range of approximately 1250 nm to 2100 nm, with minimal transmission, while the TM mode exhibits multiple resonant peaks with a broader transmission spectrum and no distinct bandgap. Therefore, the TE mode was chosen because it provides a well-defined photonic bandgap, which is suitable for biosensing applications. Additionally, the effects of varying the lattice constant on the photonic bandgap were investigated. Decreasing the lattice constant to 450 nm resulted in a bandgap spanning 1160 to 1970 nm (width: 810 nm), while increasing it to 640 nm shifted the bandgap to 1370–2280 nm (width: 910 nm). These results demonstrate a redshift in the bandgap position and a gradual increase in its width with increasing lattice constant.

**Fig. 3:**
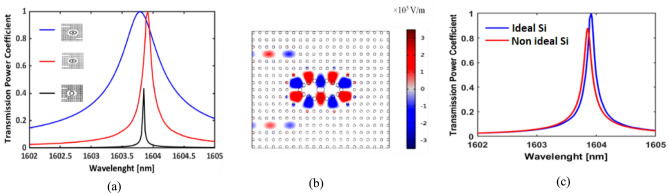
(**a**) Transmission spectra for different waveguide configurations and cavity shape, showing a high Q-factor (9270) for the optimized structure (red), a lower Q-factor (1802) for the misaligned case (blue), indicating reduced performance, and the circular cavity (black curve) exhibits lower transmission power coefficient compared to the optimized design. The inset figures illustrate the corresponding structural layouts, (**b**) Electric field distribution (Ez component) of the optimized waveguide at 1604 nm. (**c**) Impact of the imaginary part of silicon’s refractive index on the transmission spectra, showing peak broadening and a Q-factor reduction due to absorption losses.

The proposed biosensor is designed using a systematic two-phase method to achieve optimal functionality. The initial phase consists of choosing two-line defects to facilitate effective signal transfer propagation with a high Q-factor, essential for enhancing the sensor’s resolution and overall effectiveness. The input waveguide is carefully engineered to guide the optical signal toward the output waveguide while ensuring robust interaction with the analyte sensing area. Figure 3a illustrates the transmission spectra, emphasizing different scenarios that illustrate how changes in the position of the output waveguide affect light transmission. In one scenario, the guided mode arrives at the output port with an impressive Q-factor of 9270, whereas another case presents a much lower Q-factor of 1802, showcasing the effectiveness of the well-designed waveguide. Additionally, as shown in Fig. 3a, changing the cavity shape from elliptical to circular results in weaker field confinement and lower sensing performance. Figure 3b depicts the field distribution for the optimally designed waveguide, where the guided mode effectively travels to the output port. Next, we examine how incorporating the imaginary part of silicon’s refractive index affects the resonance characteristics, as shown in Fig. 3(c). The results indicate that introducing the imaginary shows peak broadening, lower transmission power, and a Q-factor reduction from 9270 to 8187. However, these slight variations suggest that the biosensor remains highly effective, even when material losses.

**Fig. 4:**
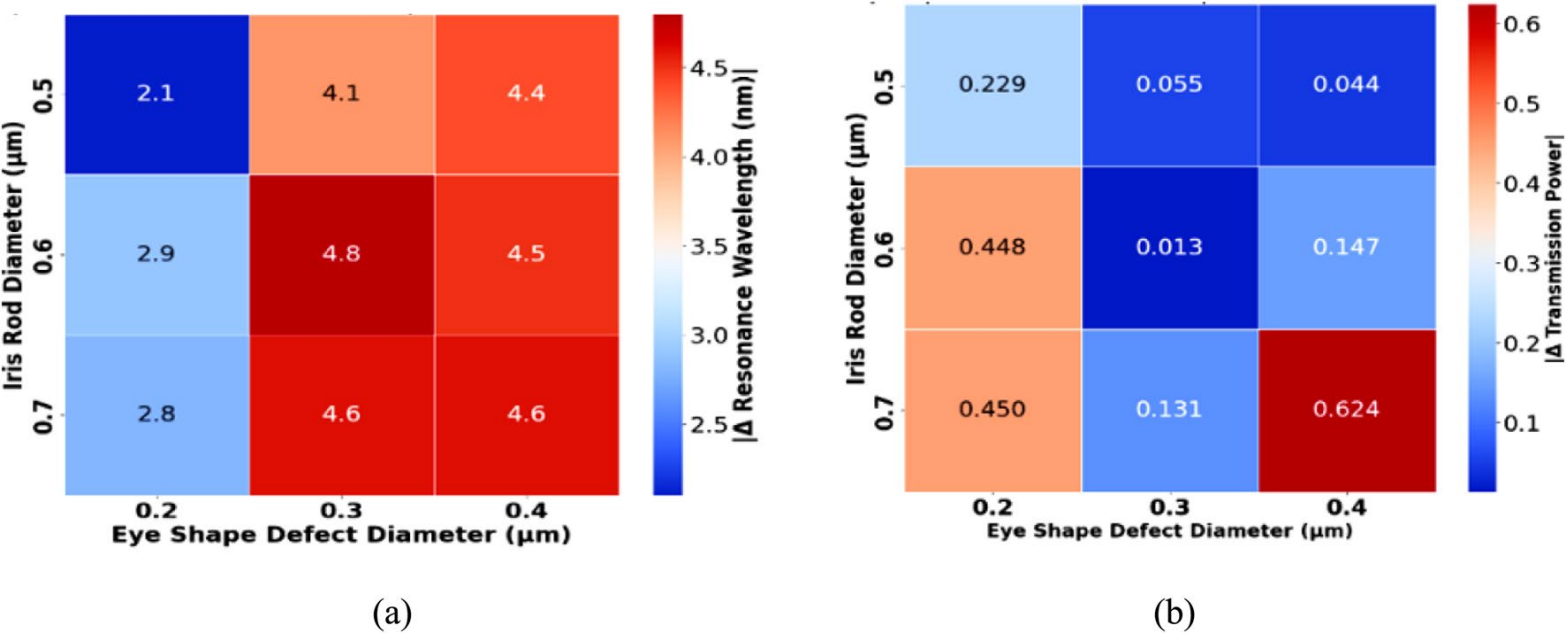
(**a**) Variation of resonance wavelength shift (Δλ) as a function of iris rod and Eye defect diameters for normal (n = 1.36) and cancerous (n = 1.38) cells. The maximum shift of (4.8) nm occurs at an iris rod diameter of 0.6 µm and an Eye-shape defect diameter of 0.3 µm, indicating the highest sensitivity. (**b**) Change in transmitted power coefficient for different iris rod and Eye-shape defect diameters. The lowest variation (ΔT) of the transmission coefficient of (0.013) is observed at the same optimal dimensions (0.6 µm, 0.3 µm), ensuring minimal power loss.

In the second phase, parametric analysis identifies optimal dimensions that maximize sensitivity while maintaining high transmission efficiency. Figures 4a and b demonstrate how changes in the diameters of both the iris rod and the Eye-shaped defect affect the performance of the sensor. In Fig. 4(a), the change in resonance wavelength (Δλ) is plotted against the diameters of the iris rod and Eye defect in response to normal cells (n = 1.36) and cancerous cells (n = 1.38). The results show that an iris rod diameter of 0.6 µm and an Eye defect diameter of 0.3 µm yield the maximum resonance shift (4.8 nm) and highest transmission coefficient (0.624), offering the best trade-off between sensitivity and transmission efficiency. This configuration optimizes the biosensor’s performance.

**Fig. 5:**
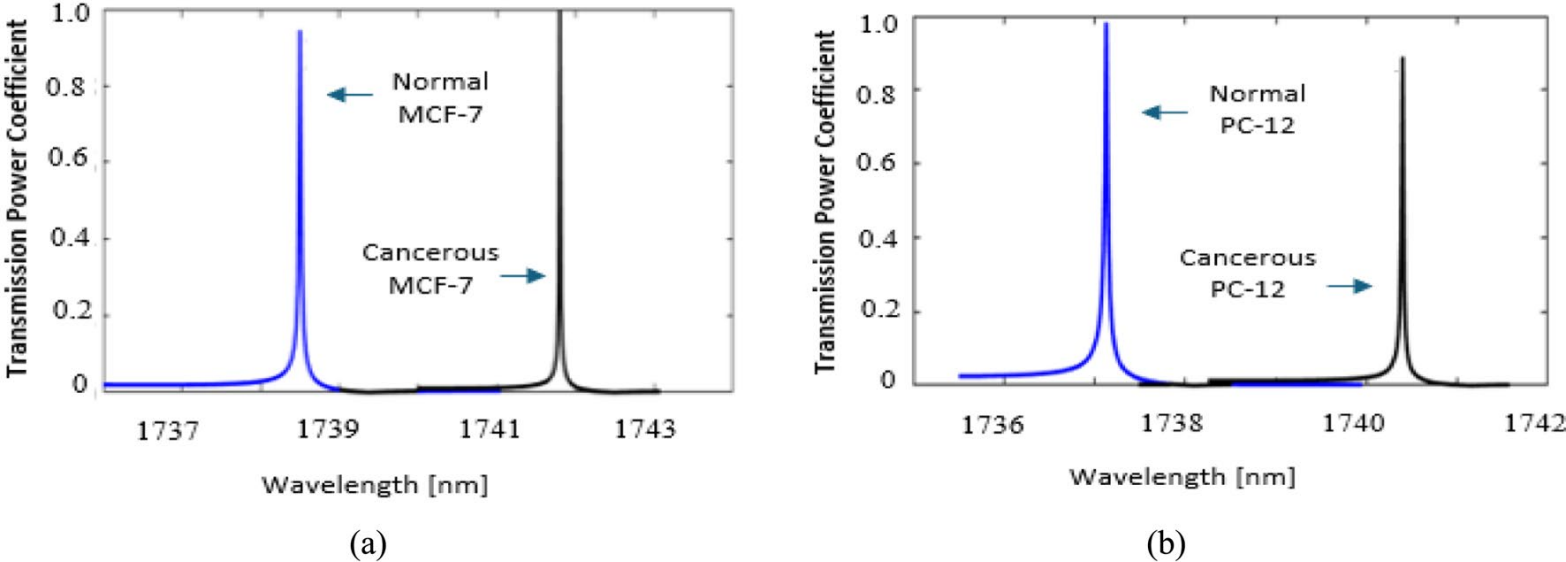
Transmission spectra of biosensor structure for identifying (**a**) MCF-7 cell, (**b**) PC12 cell.

The optimized biosensor is applied to identify various types of cancerous cells, including blood cancer (Jurkat), skin cancer (Basal), cervical cancer (HeLa), two forms of breast cancer (MCF-7 and MDA-MB-231), and adrenal gland cancer (PC12). Figure 5 shows the calculated transmitted spectra for only two cases of normal and cancerous cells: breast, MCF-7, and adrenal gland cancer PC12. The results indicate that the curves exhibit sharp and well-defined resonances with a high optical confinement and low energy loss.

The data shown in Table 2 illustrates the performance of applying the optimized biosensor to identify all types of cancerous cells. As shown in the table, the proposed PhC biosensor exhibits sensitivity levels that fall within acceptable ranges, with a peak value of 243 and a low of 236, and a FoM that fluctuates between 4800 and 11,800. Furthermore, the Q-factor values span from a high of 87,070 to a low of 15,764. The results prove the visibility of the optimized Eye-shape defect in enhancing the Q-factor by creating localized modes, concentrating light within a smaller volume, and reducing scattering losses.

**Table 2:** Biosensor performance in identifying: Basal, MCF-7, MDA-MD-231, PC12, HeLa, and Jurkat cells**.**

Type of Cells	RI	Q (unitless)	S(nm/RIU)	FoM (RIU^-1^)
Normal cell	1.36	15,746	240	4800
Basal / Skin Cancer	1.38	34,738		
Normal cell	1.387	86,925	236	7866.6
MCF-7 / Breast Cancer	1.401	58,060		
Normal cell	1.385	34,760	243	8101.5
MDA-MB-231/ Breast Cancer	1.399	58,047		
Normal cell	1.381	43,428	236	11,800
PC12 / Adrenal Glands Cancer	1.395	87,020		
Normal cell	1.368	19,267	242	6050
HeLa / Cervical Cancer	1.392	43,495		
Normal cell	1.376	34,716	243	6075
Jurkat / Blood Cancer	1.39	43,480		

**Fig. 6:**
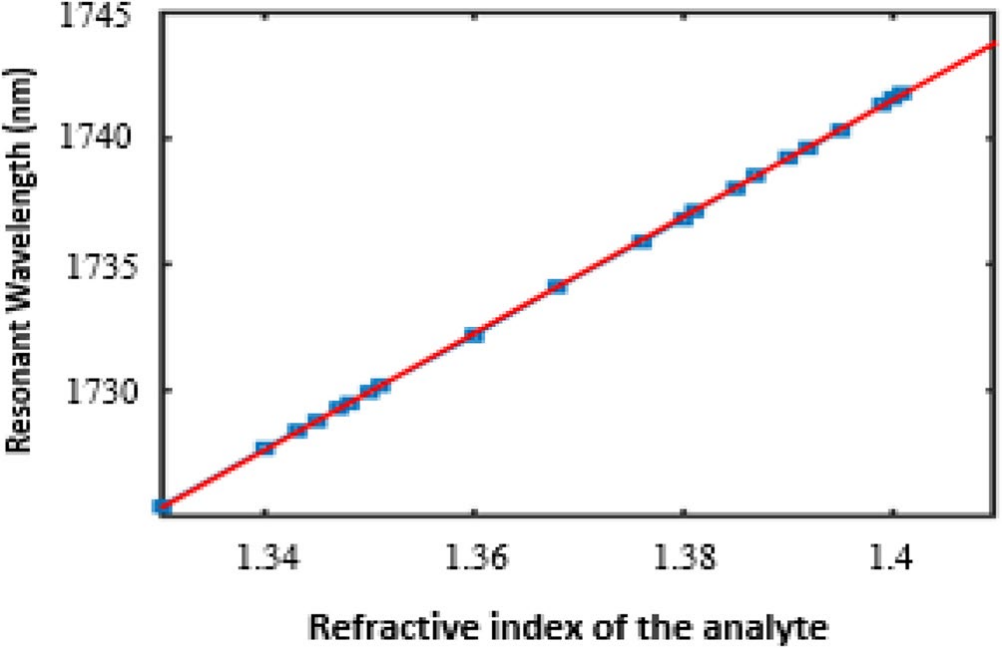
shows the relationship between resonant wavelengths and changes in refractive index.

A crucial characteristic of high-performance sensors is their linear response. To explore this further, Fig. 6 shows the relation between the refractive index of the analyte n and the resultant resonant wavelength* λ*. The relation is linear and is given by

5 $$\uplambda =230.5\text{n}+1419$$

The slope of 230.5 indicates the sensor’s average sensitivity. This linear behaviour facilitates accurate quantitative detection of refractive index variations, rendering the biosensor well-suited for precise clinical diagnostics. Moreover, it enables robust calibration strategies that address challenges posed by complex biological matrices (e.g., blood plasma), ensuring consistent and reliable performance beyond controlled laboratory environments.

Table 3 presents a comparative assessment of the performance of our proposed biosensor concerning previously reported designs, highlighting important parameters such as sensitivity, Q-factor, and overall effectiveness in identifying cancerous cells. The findings reveal noteworthy improvements, especially for cancerous cells like MCF-7 and PC12, where our biosensor achieves an astonishingly high Q-factor of up to 87,025, significantly exceeding prior studies, which generally report values under 5000. In addition, our sensor exhibits exceptional sensitivity across various cancer types, reaching as high as 243 nm/RIU, coupled with an impressive FoM of 11,800 RIU⁻^1^, signifying enhanced selectivity and detection efficacy. These advancements highlight how our design effectively improves the detection and differentiation of cancer cells.

**Table 3:** Comparison between the proposed biosensor and previously reported designs.

Ref	Normal/Abnormal Cells	S (nm/RIU)	Maximum Q-factor	FoM (RIU^-1^)
^34^	Basal	189	2542	NA
	MCF-7	320	3690	NA
	MDA-MB-231	324	3404	NA
	PC12	269	3401	NA
	HeLa	255	3012	NA
	Jurkat	263	3080	NA
^33^	Basal	100	2629	172.35
	MCF-7	92.85	262	16
	MDA-MB-231	214.28	276	38.88
	PC12	71.24	271	12.73
	HeLa	66.66	2241	97.99
	Jurkat	71.24	3049	142.83
^13^	Basal	645	4882	2224
	MCF-7	664	4122	1897
	MDA-MB-231	678	4650	2187
	PC12	664	4611	2142
	Jurkat	657	4601	2119
^41^	MCF-7	900	164	100
	MDA-MB-231	725	164	90
	PC12	725	160	90
	HeLa	725	140	80
	Jurkat	710	185	103
This work	Basal	240	34738	4800
	MCF-7	236	58060	7866.6
	MDA-MB-231	243	58047	8101.5
	PC12	236	87020	11800
	HeLa	242	43495	6050
	Jurkat	243	43480	6075

**Fig. 7:**
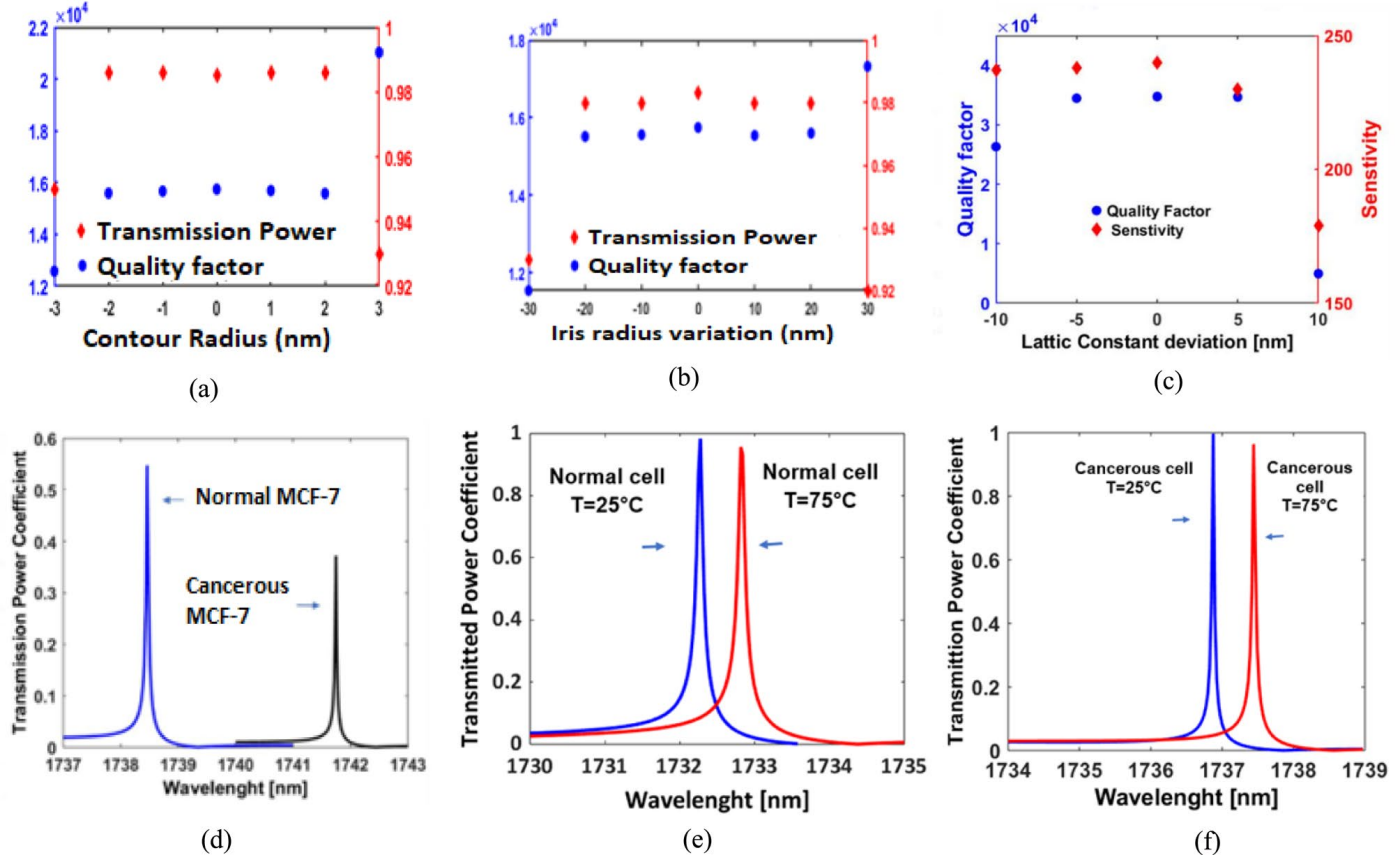
Fabrication Tolerance (**a**) variation of Contour radius, (**b**) variation of Iris radius in the range of ± 20 nm, (**c**) Effect of lattice constant variation on the sensor performance, (**d**) Impact of the imaginary part of silicon’s refractive index on the transmission spectrum, for MCF-7, (**e**) Resonance wavelength response of the biosensor at two different temperatures (25°C and 75°C) for normal condition, (**f**) Resonance wavelength response of the biosensor at two different temperatures (25°C and 75°C) for cancerous basal conditions.

To ensure the reliable performance of the proposed PhC biosensor, it is essential to evaluate its fabrication tolerance under various conditions. Three key factors that can influence the sensor’s efficiency and accuracy are geometric parameter variations, the imaginary part of silicon’s refractive index, and temperature fluctuations. Understanding the impact of these factors helps in assessing the robustness of the biosensor and its capability to maintain high sensitivity and stability under real-world fabrication and operational conditions. First, we have analyzed the effects of the geometric variations in the iris radius and contour radius on the normalized transmitted power coefficient and Q-factor. As illustrated in Figs. 7a, and 7b, deviations within a range of ± 2 nm, ± 20 nm in the contour radius, iris radius, respectively, exhibit minimal impact on the sensor’s transmission efficiency and Q-factor. Additionally, Fig. 7c shows the influence of lattice constant variations on the performance of the sensor. The results show that within a ± 5 nm deviation in lattice constant, sensitivity (230–240) and Q-factor (34,469–34,738) remain relatively stable, with maximum errors of 4.16% and 0.77%, respectively. These minor deviations confirm the sensor’s fabrication tolerance and consistent optical performance. Moreover, the suggested biosensor’s performance was assessed using non-ideal silicon with a complex refractive index as shown in Fig. 7d. The results showed no significant differences in sensitivity when compared to the ideal condition. Specifically, sensitivity slightly decreases from 236 to 233, showing that our biosensor remains highly effective, even when material losses are considered. Finally, the role of temperature variations has been investigated. The thermal stability of the proposed Eye-shaped PhC biosensor is another critical factor for practical applications. Since temperature variations can alter the refractive index of materials and shift the resonance wavelength, minimizing these effects is essential. Using silicon in our design helps mitigate thermal sensitivity due to its relatively low thermo-optic coefficient (TOC) of approximately 1.5 × 10⁻^4^ K⁻^1^ in the near-infrared range (NIR)^42^. To ensure the thermal stability through simulation, Figs. 7(e, f) show that the biosensor maintains excellent performance between 25°C and 75°C, with only slight resonance wavelength shifts and minimal sensitivity change (from 240 to 233 nm/RIU), confirming reliable performance under varying temperature conditions. Unlike Bloch Surface Wave (BSW) sensors^43^ , which are highly sensitive to temperature fluctuations, the proposed sensor operates in a more thermally stable regime.

Additionally, biosensing applications often take place in controlled environments, further reducing the impact of external temperature variations. If necessary, temperature compensation techniques, such as calibration and thermal stabilization, can further enhance the sensor’s performance.^44^. Thermal stabilization, achieved through a Peltier module, minimizes shifts in resonance due to silicon’s high TOC. Together, these processes enhance measurement precision, improve detection limits for refractive index changes, and enable multiparametric biochemical analysis. Previous studies have shown that factors like material composition and porosity also influence the thermal behaviour of PhC sensors, making careful material selection essential for ensuring stability^42^^.^ Furthermore, the suggested biosensor is built on PhC, a well-established platform with proven fabrication capabilities, therefore, manufacturing will not be a major barrier.

Advanced fabrication techniques have been recommended to achieve perfect alignment and patterning, including focused ion beam (FIB) milling^45^ , electron beam lithography (EBL)^50^, and deep reactive ion etching (DRIE)^51^. These cutting-edge nanofabrication processes allow for exceptional precision in sub-wavelength features such as silicon rods and an Eye-shaped cavity. Additionally, the biosensor operates in the NIR range, enabling easy integration with silicon photonics, Photonic Crystal Fiber (PCF), and SOI-based lab-on-chip platforms. The proposed PhC biosensor fabrication process^46^ begins with selecting silicon rods as the core material, followed by choosing a suitable nanofabrication technique—either a bottom-up method like self-assembly or a top-down approach such as EBL^47^ or NIL^45^. After fabrication, surface treatments may be applied to enhance structural or optical performance. The PhC is then integrated with biosensing components for biological detection, and the final device undergoes testing and optimization. It worth mentioned that fabricating Eye-shaped cavities using EBL^47^ and FIB milling^45^ is challenging due to complex geometries and high-resolution requirements. EBL may experience proximity effects and pattern distortion^48^, along with issues like line-edge roughness and collapse in curved features^49^. FIB offers precision but can cause surface damage and material redeposition^50^Alignment errors also risk structural distortion. To overcome these issues, techniques such as Proximity Effect Correction (PEC)^48^ high-resolution resists and multi-pass exposure^48^,low-current or gas-assisted FIB milling^51^, and precise alignment using markers^51^ are employed to enhance fabrication accuracy for complex photonic structures. The proposed Eye-shaped biosensor is tailored for real-world clinical applications, particularly for detecting cancerous cells in complex biological samples such as blood and tissue. While cancerous cells (10–30 µm) exceed the cavity’s sensing region, refractive index variations in a liquid medium remain an effective sensing mechanism^32^. Additionally, exosomes (30–150 nm) serve as crucial cancer biomarkers containing disease-specific proteins, RNA, and lipids, offering a non-invasive diagnostic option. Given their nanoscale nature, a biosensor designed to detect particles around 300 nm is feasible, provided it targets extracellular vesicles rather than whole cells. Exosomes can be obtained from various cancer cell lines, including MCF-7, MDA-MB-231, PC12, HeLa, and Jurkat, using established isolation techniques such as ultracentrifugation, size-exclusion chromatography (SEC), polymer-based precipitation (ExoQuick), and immunoaffinity isolation via CD63, CD9, and CD81 markers. Additionally, to enhance specificity and minimize cross-reactivity, selective receptors like antibodies or aptamers can be employed^52^, along with polyethylene glycol (PEG) coatings to reduce nonspecific adsorption and improve signal clarity^53^. Effective sample preparation is crucial for clinical use, with filtration, centrifugation, and microfluidic-based pre-treatment modules ensuring sample consistency^54^. The biosensor’s high sensitivity and selectivity, enabled by strong light confinement in the PhC cavity, allows for biomarker detection at low concentrations, with tunable resonance shifts optimizing detection thresholds for medical diagnostics.

**Fig. 8:**
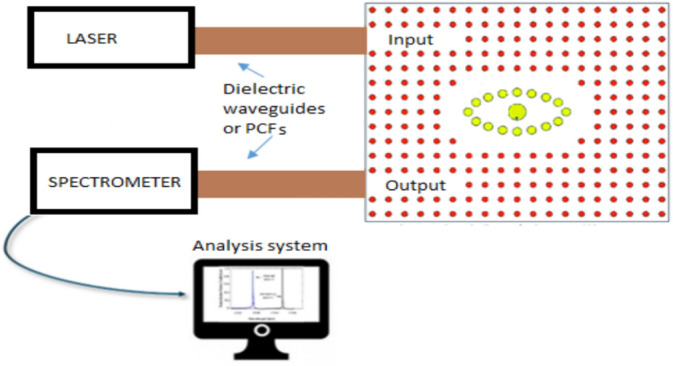
Schematic of the proposed complete optical sensing system for cancer detection.

Figure 8 presents a schematic of the proposed complete optical sensing system. The sensor consists of a square array of silicon rods, with an upper line defect serving as the input, excited by a laser source through an optical dielectric waveguide or PCF. The positioning and alignment of the input and output waveguides relative to the PhC are critical for efficient mode coupling. Lateral and angular alignment strategies are often employed to maximize mode overlap and minimize scattering losses at the interface. Techniques such as direct butt-coupling, grating couplers, and lensed fibers are commonly used for light injection into the waveguide^55^. Additionally, the waveguide is often tapered to match the mode profile of the PhC line-defect waveguide, enhancing optical confinement and reducing reflection^56^. At the center, receptor rods selectively interact with specific biomolecules, modulating the transmitted signal. The output signal collected from the lower line defect is analyzed by a spectrometer, with results displayed on a computer for further processing and interpretation.

This system is part of a proposed design and is subject to further validation and development. To improve accuracy and reduce noise in the resonance wavelength, especially when detecting small refractive index changes, techniques such as baseline correction, normalization, and curve fitting (e.g., Gaussian or Lorentzian)^57^ are used. Digital filtering^58^ helps stabilize signals, while temperature compensation algorithms correct thermal shifts in real time^59^. The proposed Eye-shaped PhC biosensor detects highly sensitive biomarkers for diseases such as cancer and diabetes. Its ultra-high Q-factors improve clinical diagnostics, and connection with lab-on-a-chip technologies enables real-time, cost-effective sensing. Its versatility contributes to tailored therapy and pharmacological research.

## Conclusion

This paper explores a highly effective PhC biosensor developed for the accurate and sensitive detection of various cancer cells. The PhC structure used displays a square-lattice configuration of silicon rods with a radius of 0.1 µm and shows a photonic bandgap ranging from 1.2 to 2.1 µm. The proposed design for the biosensor includes two-line defects that act as input and output waveguides, as well as an Eye-shaped cavity that holds the analyte in the form of rods. These rods are strategically positioned along the Eye-shaped edge and the center, which resembles an iris, enabling precise detection through shifts in the resonance wavelength caused by variations in the refractive index. The proposed biosensor demonstrates outstanding performance in detecting cancer cells with a high Q-factor, acceptable sensitivity, high FoM, and high transmission power coefficient of 87,020, 243 nm/RIU, 11,800 RIU, and 99.9%, respectively. Furthermore, the proposed biosensor exhibits a linear response and demonstrates better outcomes than those reported in the literature, maintaining exceptional performance even with material absorption losses or manufacturing defects of up to 20 nm, thus offering a dependable foundation for medical applications that demand precise biosensing functions.

## Data Availability

All data will be available upon request from [Nihal F. F. Areed, nahoolaf@mans.edu.eg].
